# Optical Slot-Waveguide Based Biochemical Sensors

**DOI:** 10.3390/s90604751

**Published:** 2009-06-16

**Authors:** Carlos Angulo Barrios

**Affiliations:** Instituto de Sistemas Optoelectrónicos y Microtecnología (ISOM), ETSI Telecomunicación, Universidad Politécnica de Madrid, Ciudad Universitaria s/n, 28040 Madrid, Spain; E-Mail: carlos.angulo.barrios@upm.es; Tel.: +34 91 5495700; Fax: +34 91 4533567

**Keywords:** integrated optics, slot-waveguides, optical sensors, biosensors

## Abstract

Slot-waveguides allow light to be guided and strongly confined inside a nanometer-scale region of low refractive index. Thus stronger light-analyte interaction can be obtained as compared to that achievable by a conventional waveguide, in which the propagating beam is confined to the high-refractive-index core of the waveguide. In addition, slot-waveguides can be fabricated by employing CMOS compatible materials and technology, enabling miniaturization, integration with electronic, photonic and fluidic components in a chip, and mass production. These advantages have made the use of slot-waveguides for highly sensitive biochemical optical integrated sensors an emerging field. In this paper, recent achievements in slot-waveguide based biochemical sensing will be reviewed. These include slot-waveguide ring resonator based refractometric label-free biosensors, label-based optical sensing, and nano-opto-mechanical sensors.

## 1. Introduction

Optical sensors are remarkable tools for analyte detection in biochemical, health and environmental applications. The use of photons for sensing makes possible multi-dimension (intensity, wavelength, phase, and polarization) and remote interrogation, immunity to electromagnetic interferences, multiplexed detection, and availability of well-established technologies from communication industries: e.g. lasers of almost any wavelength, detector arrays, micro-/nano-machining, waveguides, and high speed links. In addition, optical frequencies coincide with a wide rage of physical properties of bio-related materials in nature.

Optical biosensing can be carried out by using two different detection strategies [1]: labeling-based detection and label-free detection. In the former protocol, either target molecules or biorecognition molecules are labeled with either fluorescence or light absorbing markers in order to detect and quantify the presence of a specific sample molecule of interest. In the label-free protocol, the target molecules are not labeled or modified, and their presence is revealed by methods such as refractometry, Raman spectroscopy and optical detection of mechanical deflection of movable elements (e.g. a cantilever).

Both labeling-based and label-free sensing schemes can be implemented by using integrated optical devices based on planar waveguides. These possess important advantages as compared to bulk optic elements and fiber optic based biochemical sensors. Planar technology facilitates fabrication by using standard lithographic techniques, allowing mass production (low cost) and integration of arrays of compact devices on a single-chip for the simultaneous detection of several analytes. In addition, mature Si-based materials and processes—CMOS technology—can be employed, which adds the capabilities of sensor integration with electronics on the same chip and sensor miniaturization due to the high refractive-index-contrast available in Si-based CMOS-compatible materials [2].

Conventional strip and rib waveguides are commonly used in biochemical sensors based on integrated optics. In these waveguides, the guiding mechanism is based on total internal reflection (TIR) in a high-index material (core) surrounded by a low-index material (cladding); the TIR mechanism can strongly confine light in the high-index material. On the other hand, there are also planar waveguides non-based on TIR, such as hollow-core waveguides [3], which are employed to guide light in low-index materials. This is especially interesting for biochemical sensing since the hollow-core can be filled with low-index fluids. However, in these guides, optical interference is involved and therefore they are highly wavelength dependent.

**Figure 1.**
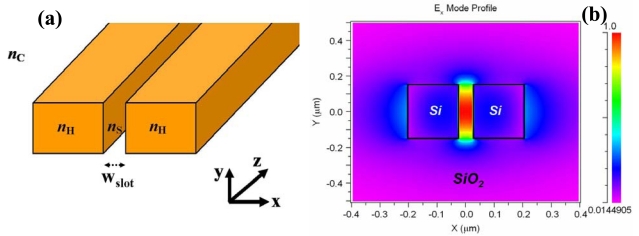
(a) Schematic view of a slot-waveguide. (b) Calculated E_x_ profile of the quasi-TE eigenmode in a Si (n_H_ = 3.45)/SiO_2_ (n_S_ = n_C_ = 1.44) slot-waveguide at a wavelength of 1.55 μm. E-field is enhanced in the nanoscale slot-region of refractive index n_S_.

A novel guided-wave configuration, known as a slot-waveguide, was introduced by Almeida *et al.* in 2004 [4]. This structure is able to guide and strongly confine light in a nanoscale low-refractive-index material by using TIR at levels that cannot be achieved with conventional waveguides. Figure 1(a) shows a schematic picture of a slot-waveguide. It consists of two strips (rails) of high refractive index (n_H_) separated by a low-index (n_S_) region (slot) of width w_slot_. The principle of operation of this structure is based on the discontinuity of the electric (E) field at a normal boundary between two materials. For an electromagnetic wave propagating in the z direction (see Figure 1), the major E-field component of the quasi-TE eigenmode (which is aligned in the x-axis) undergoes a discontinuity at the perpendicular rails/slot interfaces that, according to Maxwell's equations, is determined by the relation |E_S_/E_H_| = (n_H_/n_S_)^2^, where S and H denote slot region and high-index region, respectively. Thus if n_H_ is much larger than n_S_, this discontinuity is such that the E-field is much more intense in the low-index slot region than in the high-index rails. Given that the width of the slot is comparable to the decay length of the field, the E-field remains high across the slot [see Figure 1(b)], resulting in a power density in the slot that is much higher than that in the high-index regions. This unique characteristic makes the slot-waveguide very attractive for numerous applications, including biochemical sensing. Using the slot as sensing region, larger light-analyte interaction, and hence higher sensitivity, can be obtained as compared to a conventional waveguide. In addition, since TIR mechanism is employed, there is no interference effect involved and the slot-structure exhibits very low wavelength-sensitivity.

In this paper, recent progress in slot-waveguide based biochemical sensors is reviewed. In Section 2 label-free refractometric biochemical sensors are presented and discussed, with emphasis on slot-waveguide microring resonators. Section 3 deals with labeling-based optical biosensing configurations. In Section 4, opto-mechanical transducers based on slot-waveguides for biochemical sensing are overviewed. Finally, conclusions are collected in Section 5.

## 2. Slot-Waveguide Based Refractometric Sensors

Refractive index (RI) sensors based on planar waveguides play a prominent role in chemical and biochemical analysis [5]. This label-free sensing method can provide real-time results with minimal sample preparation. In these sensors, a fraction of a guided optical probe interacts with the sample to be tested. A change in RI of the probed region causes a corresponding phase shift that can be detected as either a frequency or an intensity shift, which is converted to the sensing signal. For applications requiring the analysis of a liquid sample (bulk or homogenous sensing), the sensing signal can be employed to determine the RI of the sample as compared to a reference sample. For biomolecule detection applications (surface sensing), the specific capture of biomolecules at the sensor surface results in a local change in RI which produces a sensing signal that enables quantification of the biomolecules in the sample.

Guided-wave planar optical RI sensors are commonly implemented by means of interferometric configurations such as directional couplers [6], Mach-Zehnder interferometers [7] and microresonators [8-11]. In particular, RI sensors based on slot-waveguide microring resonators [12-15] and directional couplers [16] have been recently demonstrated and proposed, respectively. The former were fabricated in two different material systems, Si_3_N_4_/SiO_2_ [12,13] and Si/SiO_2_ [14,15]. In this section, these slot-waveguide based RI sensor configurations are reviewed, with stress on microring-resonator sensors since they have been experimentally tested.

### 2.1. Silicon nitride slot-waveguide microring resonator based sensors

The first experimental demonstration of a slot-waveguide based biochemical sensor was achieved by Barrios *et al.* for both bulk [12] and surface sensing [13]. These authors employed a vertical (slot/rail interface is normal to the substrate) slot-waveguide ring resonator made of silicon nitride on silicon dioxide. The use of Si_3_N_4_ as high-index material (instead of higher-index Si) allows the definition of a wider slot region while maintaining single-mode operation [17]. The main purpose of having a wider slot region is to facilitate filling it with liquids for sensing and optofluidic applications. The device sensor was probed at a wavelength around 1.3 μm, which is typically used in telecomm applications (O-band) and leads to lower water optical absorption than that at the other common telecom wavelength, 1.55 μm.

**Figure 2.**
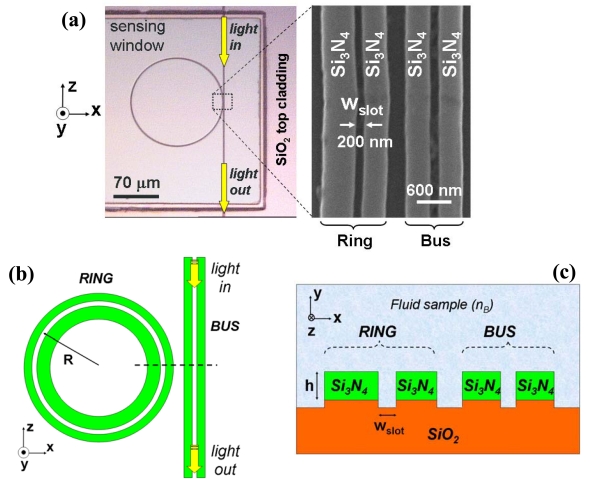
(a) Left: Top view photograph of a 70-μm-radius Si_3_N_4_ slot-waveguide microring resonator. Right: Scanning electron microscope image of the coupling region. (b) Schematic top view. (c) Schematic cross-section along the dotted line [Figure 2(b)].

Figure 2(a) shows optical microscope and scanning electron microscope (SEM) images of the demonstrated Si_3_N_4_/SiO_2_ slot-waveguide ring sensor. A straight slot-waveguide (bus) was used to couple light into an asymmetric (the inner rail is wider than the outer rail) slot-waveguide ring of radius R = 70 μm. Ring-waveguide asymmetry aims to reduce ring optical losses. Si_3_N_4_ device layer was 300 nm in thickness (h) and the slot width of the ring waveguide was w_slot_ ≈ 200 nm.

**Figure 3.**
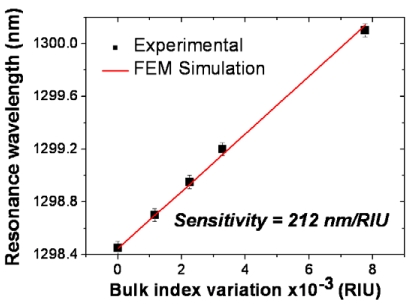
Resonance wavelength of the Si_3_N_4_ slot-waveguide ring resonator as a function of the bulk (top cladding) refractive index variation. Black dots: experimental data (water-ethanol solutions) [12]; red line: finite element method (FEM) based calculations [18].

Homogeneous sensing was studied by depositing drops of liquids (water-ethanol solutions) with varying refractive indexes (concentrations) over the ring and monitoring the corresponding red-shift of a ring resonance wavelength. The resulting bulk RI sensitivity was measured to be S_B_ = Δλ_r_/Δn_B_ = 212 nm/RIU (see Figure 3), where Δλ_r_ is the resonance wavelength shift and Δn_B_ is the bulk (fluid) index variation. This sensitivity is more than twice larger than that exhibited by biochemical sensors based on conventional-waveguide microrings [9,10], indicating that higher analyte-light interaction occurs in the slot-waveguide sensor. The device bulk sensitivity was also theoretically estimated [18] by using different numerical methods – to calculate the effective index variation (Δn_eff_) of the ring optical mode as a function of Δn_B_ – and the wavelength tuning relationship for microresonators:

(1) $$\Delta \lambda_{r}=-\Delta n_{\text{eff}}\frac{2\pi }{\lambda_{r0}}\cdot {\left(-\frac{2\pi n_{g}(\lambda_{r0})}{\lambda_{r0}^{2}}\right)}^{-1}=\lambda_{r0}\frac{\Delta n_{\text{eff}}(\Delta n_{B},\Delta \lambda_{r})}{n_{g}(\lambda_{r0})}$$

where n_g_(λ_r0_) is the group index, which is defined as n_g_ = n_eff_ - λ(∂λ/∂n_eff_), at the unperturbed (Δn_B_ = 0) resonance wavelength λ_r0_. As seen in Figure 3, excellent agreement was found between the calculated and measured sensitivities [18,19], suggesting that the slot ring channel was fully filled with the different aqueous solutions employed in the measurements. This is a remarkable conclusion because it demonstrates feasibility of the Si_3_N_4_ slot-waveguide ring device to be used in optofluidic applications where enhanced light-liquid interaction within the nanoscale slot region is desired.

**Figure 4.**
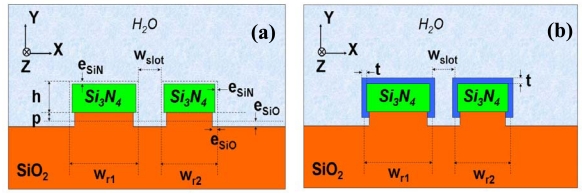
Schematic cross-sections of the ring slot-waveguide for (a) HF surface etching and (b) adlayer surface sensing [18]. w_r1_, w_r2_, w_slot_, h, and p are the initial device dimensions (before HF etching). e_SiN_ and e_SiO_ are the HF etching depths in Si_3_N_4_ and SiO_2_, respectively, and t is the adlayer thickness.

Device surface sensitivity was characterized by studying the adhesion of biomolecules to the Si_3_N_4_ slot-waveguide ring resonator [13]. First, the device was treated with highly diluted (1%) hydrofluoric acid (HF) in order to produce oxide-free and amine-containing silicon nitride surfaces and thus to allow functionalization of the waveguide rails by means of selective immobilization of glutaraldehyde molecules on their surfaces. HF solution slightly etched both Si_3_N_4_ and SiO_2_ materials, altering the slot-waveguide dimensions, as illustrated schematically in Figure 4(a), and decreasing the effective index of the optical mode in the ring. As a consequence, a blue-shift of the ring wavelength resonances was observed [18]; that is, the device behaved as an HF optical sensor based on surface etching. After this HF treatment, the device was successively covered with glutaraldehyde molecules, anti-BSA (bovine serum albumin) antibodies and BSA antigens demonstrating the capability of the sensor to carry out immunoassays. The device showed a protein detection limit of ˜20 pg/mm^2^. Biomolecule coverage of the slot-waveguide is shown schematically in Figure 4(b) and was modeled in [18].

**Figure 5.**
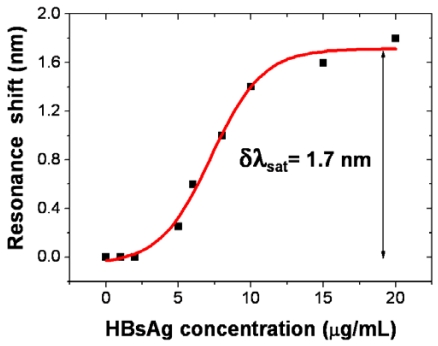
Resonance wavelength shift of a 70-μm-radius Si_3_N_4_ slot-waveguide ring resonator as a function of hepatitis B surface antigen (HBsAg) concentration [20]. Square dots: experimental data; red line: sigmoidal fit.

Recently, similar surface sensing experiments have also demonstrated immobilization of hepatitis B surface antigen (HBsAg) molecules on a Si_3_N_4_ slot-waveguide ring sensor [20]. Figure 5 shows the resonance wavelength shift of the microring resonator as a function of the concentration of HBsAg. It is seen that the experimental data obey a sigmoidal relation (fit curve in Figure 5). At high concentrations the resonance shift saturates indicating that the sensor surface is fully covered with antigens.

**Figure 6.**
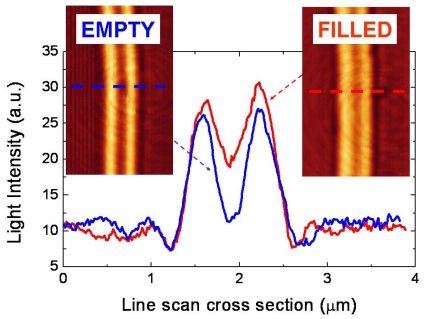
Confocal microscope intensity profiles of a Si_3_N_4_ slot-waveguide before isopropanol droplet deposition (blue line) and after isopropanol droplet evaporation (red line). Intensity profiles were taken along the dotted lines shown in the corresponding laser scanning confocal microscope images. Courtesy of M. Holgado (Centro Láser-Universidad Politécnica de Madrid).

Another Si_3_N_4_ slot-waveguide microring resonator was also used to demonstrate optofluidic device-reconfigurability [21]. It was observed that small amounts of organic liquids were trapped, due to capillary and wetting forces, inside the slot-nanochannel, modifying dramatically the microring resonator optical response. The presence of stored liquid inside the slot-nanotrench was also studied by using laser scanning confocal microscopy. Figure 6 shows confocal microscope images of the Si_3_N_4_ ring slot-waveguide before depositing a droplet of isopropanol (empty slot) and several hours after bulk evaporation of a deposited isopropanol droplet. The confocal microscope intensity profiles indicate the presence of adhered liquid inside the slot after isopropanol bulk evaporation. Trapped liquid can be removed by applying heat or chemical cleaning. Potential optofluidic applications of this effect include permanent and rewriteable photonic configurations, process monitoring, *in-situ* chemical detection, and study of liquid-solid interfacial forces at the nanoscale.

To date, the latest achievement in Si_3_N_4_ ring-resonator sensors has been the integration of a Si_3_N_4_ microring array with a PDMS microfluidic network on a Si single chip, enabling accurate multiplexed assays in labs-on-chip [22]. In addition, improvements in the device fabrication have enabled to increase the bulk RI sensitivity of these Si_3_N_4_ slot-waveguide ring sensors up to 246 nm/RIU.

### 2.2. Silicon slot-waveguide microring resonator based sensors

The use of high index-contrast material system, such as Si/SiO_2_, enables to reduce photonic-device dimensions and to increase the E-field enhancement at the slot/rails interfaces in slot-waveguides, that is, to increase the sensor sensitivity, as compared to moderate index-contrast material systems, such as Si_3_N_4_/SiO_2_. Dell'Olio *et al.* theoretically investigated silicon-on-insulator (SOI) slot-waveguides for highly sensitive and compact biochemical integrated optical sensing [23]. These authors compared the calculated sensitivities of different SOI slot-waveguide configurations with those exhibited by other silicon nanometer guiding structures, such as rib or wire waveguides. After design optimization, it was concluded that the homogeneous sensitivity of an SOI slot-waveguide is significantly larger than those achievable by other nanometer guiding structures.

On the other hand, higher index-contrast also implies higher sensitivity to device-dimension deviations and surface roughness (optical scattering losses), and, in slot-waveguides, narrower slot widths (≤ 100 nm) than those required in Si_3_N_4_/SiO_2_. The latter issue makes difficult to introduce certain liquids inside the narrow slot region of Si/SiO_2_ slot-waveguides due to capillary and surface tension considerations. However, this drawback does not exist for gases, making Si/SiO_2_ slot-waveguides ideal structures for gas sensing on-chip. Robinson *et al.* [14] implemented a slotted Si/SiO_2_ microng resonant cavity to detect small changes in the refractive index of acetylene gas due to composition and pressure. The slot width was as small as 40 nm, leading to a very high E-field enhancement in the slot region. The RI bulk sensitivity of this device was measured to be as high as 490 nm/RIU, evidencing larger light-fluid interaction than that obtained with Si_3_N_4_/SiO_2_ slot-waveguides.

Recently, Claes *et al.* [15] demonstrated label-free biosensing with a SOI slot-waveguide racetrack resonator. The slot width of the sensing waveguide was 100 nm. The bulk sensitivity of this device was measured to be 298 nm/RIU as compared to 68 nm/RIU [10] for normal (strip waveguide based) racetrack based RI sensors. Experiments with biotin/streptavidin binding demonstrated that the slot-waveguide sensor was 4.8 times more sensitive (surface sensing) than those based on racetrack resonator formed by conventional waveguides [10]. These authors also theoretically estimated the device bulk sensitivity, which resulted to be higher than the experimental value. This disagreement suggests that the slot-region was not fully filled with the aqueous solutions employed in the experiments, due to the aforementioned capillary and surface tension issues.

**Figure 7.**
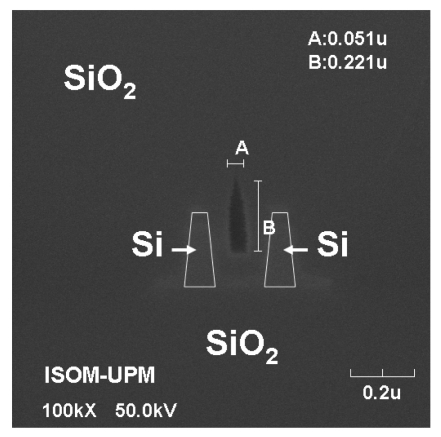
Cross-sectional SEM image of a void nanochannel obtained by plasma-enhanced chemical vapor deposition (PECVD) of SiO_2_ on a SOI slot-waveguide configuration. Courtesy of D. López-Romero (ISOM-Universidad Politécnica de Madrid).

Difficulty to fill narrow gaps in vertical slot-waveguides also ocurrs when the slot region is intended to be filled with a condensed low-index material (e.g. SiO_2_) by using common deposition techniques such as plasma-enhanced chemical vapor deposition (CVD) and low-pressure CVD. Figure 7 shows a typical result of depositing SiO_2_ by CVD on a vertical slot-waveguide configuration. A void in the slot region is formed because of non-conformal deposition. This might be harmful for applications where maximum light interaction with the deposited material is desired. However, it could be advantageously used for sensing purposes. It has been theoretically shown that the E-field in the (air) void is actually increased as compared to a traditional slot-waveguide [24]. Thus the propagating optical mode would be significantly modified by the presence of fluids and particles (biomolecules) inside the nanopipe created along the waveguide. Due to the nanometer cross-sectional dimensions of the slot-nanochannel, extremely small amounts of analytes would be required to produce a significant sensing signal. Although the injection and flow control of fluids and molecules in the slot-nanopipe might be challenging from a practical point of view, research on this subject would enable one to carry out studies on simultaneous confinement of light and fluids at the nanoscale, and take advantage of the phenomenon in order to implement high performance nano-opto-fluidic devices and architectures based on slot-waveguides.

### 2.3. Other slot-waveguide based RI sensor configurations

**Figure 8.**
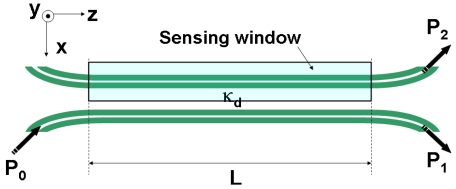
Schematic top view of a directional coupler formed by slot-waveguides. Variation of the refractive index in the sensing region alters both P_1_ and P_2_ output powers. κ_d_ is the coupling coefficient between the slot-waveguides.

In addition to microring resonators, other interferometric configurations, such as Mach-Zenhder interferometers, directional couplers, and Fabry-Perot cavities, could also work as highly sensitive transducers for RI label-free sensing. Both, passive directional couplers [25] and Fabry-Perot cavities [17] based on slot-waveguides have been demonstrated in Si/SiO_2_ and Si_3_N_4_/SiO_2_, respectively, and they could be used for RI sensing by defining a proper sensing window on them. Passaro *et al.* [16] reported a theoretical analysis of a compact directional coupler formed by SOI slot-waveguides for chemical sensing similar to that schematically illustrated in Figure 8.

The minimum detectable refractive index change was calculated to be 10^-5^. Sensor design and optimization allowed a good trade-off between device length (L) and sensitivity. The estimated sensor limit-of-detection to glucose concentration change in an aqueous solution was of the order of 0.1 g/L.

**Figure 9.**
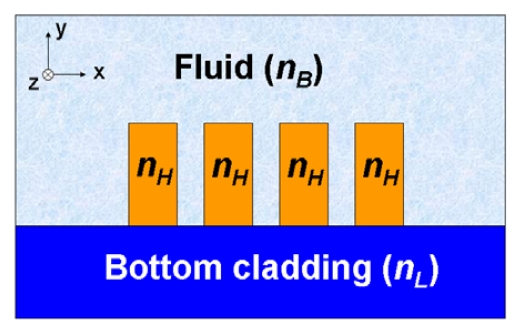
Schematic cross-section of a multi (triple) -slot waveguide. n_H_, n_B_ and n_L_ are the refractive indexes of the rails, top cladding fluid (sensing region) and bottom cladding layer, respectively, such as n_H_ > n_B_, n_L_.

In the optical configurations described so far, vertical slot-waveguides with a single slot-sensing region have been always used. However, the slot-waveguide principle can be extended to other geometries such as vertical multi-slot [26,27] and horizontal (multi-)slot waveguides [28,29]. Both types of structures have been demonstrated to operate properly as passive optical elements in Si/SiO_2_ [26,29], Si/Si_3_N_4_/SiO_2_ [28] and Si_3_N_4_/SiO_2_ [27] material systems. Figure 9 shows a schematic cross-sectional view of a multi (triple)-slot waveguide. The effective index variation of the guided mode for a Si_3_N_4_/SiO_2_ multiple (triple)-slot structure as a function of the bulk (fluid) refractive index variation has been estimated to be improved by 20% as compared to that of a single-slot waveguide [27]. This is a consequence of a larger localization of the E-field of the propagating optical mode in the sensing region. However, multi-slot structures possess more high-index interfaces, which may increase optical scattering losses due to interface roughness – arising mainly from dry etching during device fabrication– degrading the sensor performance. On the other hand, horizontal (slot/rail interface is parallel to the substrate) slot-waveguide based configurations can be implemented by using conventional deposition techniques (CVD) which results advantageous to control the slot distance and to reduce rail/slot interface roughness.

## 3. Labeling-Based Optical Slot-Waveguide Sensors

The evanescent field in planar waveguides can be employed in both optical absorption based sensors and fluorescence sensors. In the former, the presence of an absorbing label (e.g. a gold nanoparticle) within the penetration depth of the evanescent field induces an increase in the propagation loss of the guided mode. Such an increase can be related to the concentration of the target analyte. In fluorescence-based sensors, the evanescent field can be used to excite the fluorescent labels residing in the immediate region near the waveguide interface and to collect the fluorescence via back tunneling into trapped modes of the waveguide [30].

Bernini *et al.* [31] investigated theoretically both fluorophore excitation and fluorescence collection efficiencies of two-dimensional (i.e. rails are slabs) slot-waveguides. They considered excitation and collection wavelengths of 633 nm and 690 nm, respectively, and refractive indexes of 2 and 1.33 for the high-index rails and low-index slot region, respectively. Their analysis indicated that the use of a slot-waveguide can improve both the excitation and collection efficiencies with respect to a single slab waveguide due to the enhanced E-field intensity at the rail/slot interfaces. In particular, numerical calculations revealed maximum excitation efficiency of 36% by using the slot geometry (slot thickness = 160 nm) as compared to 26% of a conventional slab waveguide. The extension of this study to three-dimensional guiding configurations would provide useful information and design guide-lines for an actual implementation of labeling-based detection configurations based on slot-waveguides.

**Figure 10.**
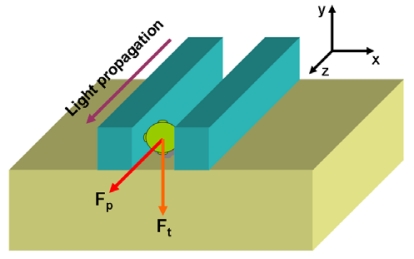
Schematic picture illustrating a nanoparticle trapped and transported by a slot-waveguide. F_t_ is the trapping force that holds the nanoparticle in the slot region; F_p_ is the radiation pressure force which transports the particle along the slot-nanochannel.

The evanescent field of waveguides extending into the surrounding liquid can also serve to trap and transport particles [32]. However, systems based on conventional waveguides are no suitable to manipulate nanometer-size particles, including biomolecules, since the particles only interact with a small portion of total transported light (most of the light is confined within the solid core of the waveguide). This limitation can be overcome by using slot-waveguides, in which the majority of the optical energy is accessible within the low-index slot region, as recently demonstrated by Yang *et al.* [33]. These authors achieved optical manipulation of polystyrene nanoparticles as small as 75 nm and dye-labeled λ-DNA molecules by using a Si/SiO_2_ slot-waveguide at 1.55 μm operation wavelength. This technique simultaneously employs near-field forces to confine matter inside the slot-waveguide and scattering/adsorption forces to transport this matter (see Figure 10). Optical manipulation based on slot-waveguides could lead to new methods of bioanalysis and directed nanoassembly.

## 4. Opto-Mechanical Slot-Waveguide Based Sensors

Micro-opto-electro-mechanical (MOEM) devices based on the principles of integrated optics and micromachining technology on Si have immense potential for sensor applications. For example, Zinoviev *et al.* demonstrated an optical waveguide microcantilever biosensor based on the sensitivity of optical power transfer between two butt-coupled waveguides to their misalignment with respect to each other [34]. Selective-coated microcantilevers are used as ultrasensitive nanomechanical sensors for molecular detection; differential surface stress due to molecular recognition leads to bending (deflection) of the cantilever, which can be detected by optical means.

**Figure 11.**
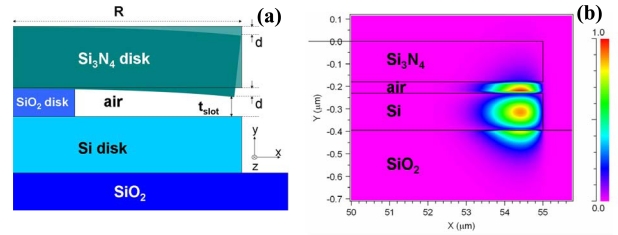
(a) Schematic cross-section of a horizontal slot-waveguide disk resonator of radius R for the detection of nanomechanical forces. Bending (d) of the Si_3_N_4_ cantilever changes the slot distance t_slot_, which in its turn changes the effective index of the optical mode in the disk. (b) Calculated TM optical mode in the horizontal slot-waveguide disk resonator for d = 0 (no deflection).

The optical performance of a slot-waveguide is very sensitive to small variations of the slot distance because of the high E-field confinement. This property has been used to propose original sensing configurations based on slot-waveguides formed by movable elements. Barrios [35] presented and analyzed a nanomechanical optical sensor consisting of a horizontal slot-waveguide integrated in a disk resonator. Figure 11(a) shows a schematic cross-section of the device. A bottom Si disk (device layer of a SOI wafer) and a top Si_3_N_4_ disk, both of them of radius R, are separated by a 50-nm-thick air gap = (t_slot_ + d), where d is the cantilever deflection. The Si_3_N_4_ layer acts as a circular cantilever supported by a smaller-radius SiO_2_ disk. As shown in Figure 11(b), the E-field distribution of the quasi-TM (major E-field is along the y-axis) optical mode is enhanced in the air slot-region. This structure is predicted to be highly sensitive to changes in optical path length produced by variations in slot distance (t_slot_) and to effective index variations of the resonator. A deflection sensitivity of 33 nm^-1^ was calculated, which is 4 orders of magnitude larger than those of state-of-the-art microcantilever sensors.

The air gap distance (t_slot_ + d) can be increased up to 100 nm by replacing the bottom Si disk by a Si_3_N_4_ disk [36]. Note that the optical principle behind this approach is similar to that described in section 2: the lower the index contrast, the wider the slot region. A larger air gap should reduce the risk of adhesion – due to capillary forces – between the movable top disk cantilever and the fixed bottom disk. As expected, the use of Si_3_N_4_ instead of Si led to lower device sensitivity; in particular, the deflection sensitivity for the Si_3_N_4_-bottom-disk device was estimated to be reduced down to 11 nm^-1^. Note, however, that this value is on the same order of magnitude as that calculated for the Si-bottom-disk device.

Other nano-opto-mechanical configurations based on slot-waveguides, such as those analyzed by Almeida *et al.* [37] consisting of Si/SiO_2_ fixed and cantilever beams, are also appealing structures to be considered as ultrasensitive transducers for biosensing.

## 5. Conclusions

In this article, the emerging field of using slot-waveguides for highly sensitive biochemical sensing has been overviewed. Hitherto, fluid (both liquid and gas) RI detection, label-free and labeling-based biosensing, trapping and transport of nanoparticles (including biomolecules) and opto-mechanical transduction based biodetection have been either demonstrated or theoretically analyzed by using CMOS-compatible material systems: Si_3_N_4_/SiO_2_ and Si/SiO_2_. Moderate index-contrast Si_3_N_4_/SiO_2_ is less susceptible to device structural imperfections and allows wider slot-widths facilitating liquid and particle infiltration inside the slot region; high index-contrast Si/SiO_2_ provides higher E-field enhancement in the slot, and therefore higher biochemical sensitivity, and enables larger integration level. In all cases, the use of slot-waveguides has been proven to be advantageous over conventional waveguides in terms of sensitivity and potential use in applications requiring the fusion of nanophotonics and nanofluidics. Future work is expected to be focused on the optimization and experimental demonstration of some of the reviewed sensing configurations, extension to other material systems, and development of new sensor architectures based on slot-waveguides for lab-on-chip platforms.
